# Supplementary material for: An Integrated In Silico, In Vitro and Tumor Tissues Study Identified Selenoprotein S (SELENOS) and Valosin-Containing Protein (VCP/p97) as Novel Potential Associated Prognostic Biomarkers in Triple Negative Breast Cancer
Source: Cancers (Basel). 2022 Jan 27;14(3):646. doi: 10.3390/cancers14030646 (PMC8833666; doi:10.3390/cancers14030646)
Supplement: Supplementary file 1 [file cancers-14-00646-s001.zip › cancers-1565701-supplementary.pdf]

cancers

MDPI

Supplementary Materials: An Integrated In Silico, In Vitro and Tumor Tissues Study Identified Selenoprotein S and Valosin-Containing Protein (VCP/p97) as Novel Potential Associated Prognostic Biomarkers in Triple Negative Breast Cancer

Susan Costantini, Andrea Polo, Francesca Capone, Marina Accardo, Angela Sorice, Rita Lombardi, Palmina Bagnara, Federica Zito Marino, Martina Amato, Michele Orditura, Maddalena Fratelli, Gennaro Ciliberto and Alfredo Budillon.

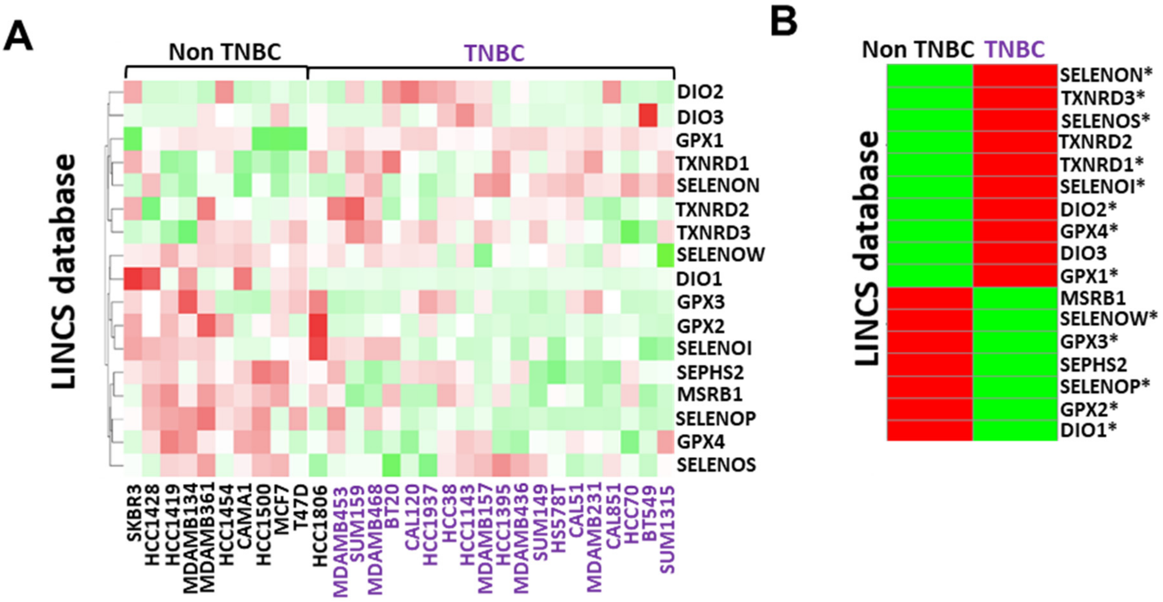

Figure S1. Comparison between the gene expression profiles of each selenoprotein in breast cancer cell lines subdivided between TNBC and “Non TNBC” sub-groups and their related mean expression profiles by clustering analysis obtained using RNAseq data in LINCS dataset (**A** and **B**). Color scale from green to red refers to lower and higher gene expression levels of selenoproteins, respectively. We evidenced with * the selenoproteins that presented similar expression trend in both CCLE and LINCS datasets.

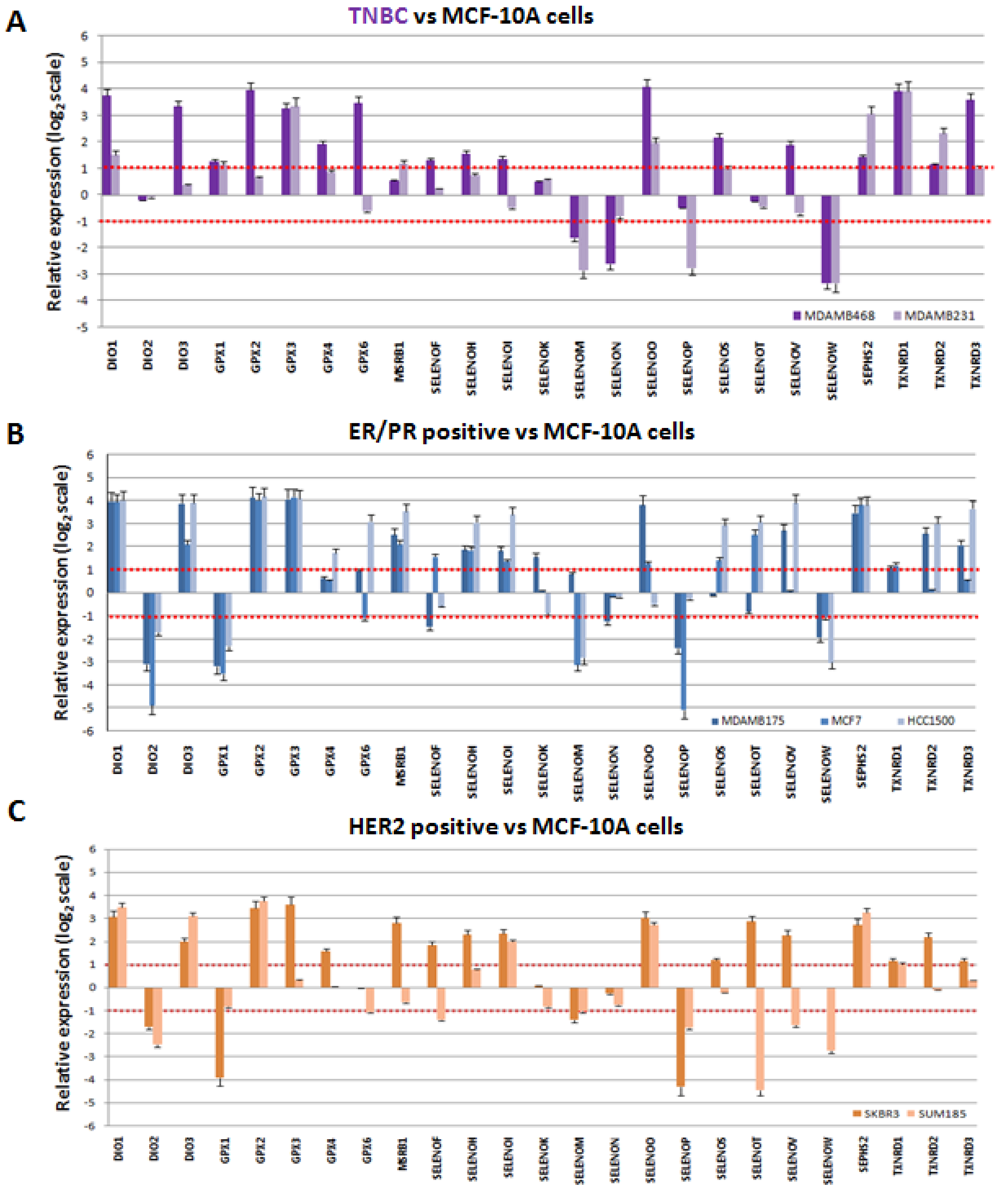
A
TNBC vs MCF-10A cells
Relative expression (log_2 scale)
MDAMB468
MDAMB231
DIO1
DIO2
DIO3
GPX1
GPX2
GPX3
GPX4
GPX6
MSRB1
SELENOF
SELENOH
SELENOI
SELENOK
SELENOM
SELENON
SELENOO
SELENOP
SELENOS
SELENOT
SELENOV
SELENOW
SEPHS2
TXNRD1
TXNRD2
TXNRD3
B
ER/PR positive vs MCF-10A cells
MDAMB175
MCF7
HCC1500
C
HER2 positive vs MCF-10A cells
SKBR3
SUM185

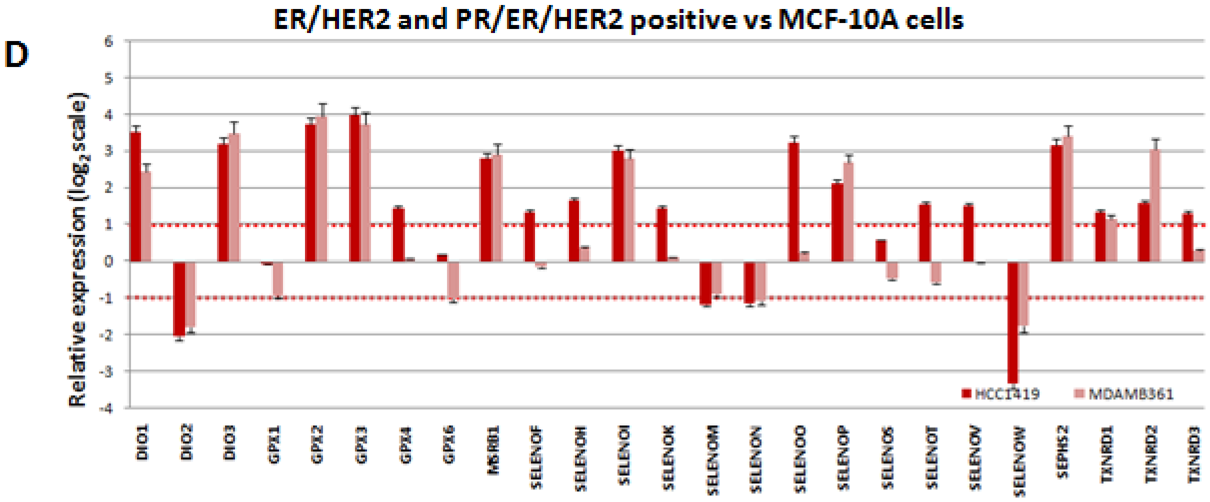

Figure S2. Fold change of gene expression levels for each selenoprotein (**A**) in two TNBC cell lines (MDAMB468 and MDAMB231), (**B**) in three ER/PR positive cells (MCF7, MDAMB175 and HCC1500), (**C**) in two HER2 positive cells (SKBR3 and SUM185), and (**D**) in two ER/HER2 and PR/ER/HER2 positive cell lines (HCC1419 and MDAMB361) compared to the non-cancerous MCF-10A cells, evaluated by $2^{-\Delta\Delta Cq}$ method and reported as $\log_2$ scale. We consider statistically significant the values higher and lower than +1 and −1, respectively.

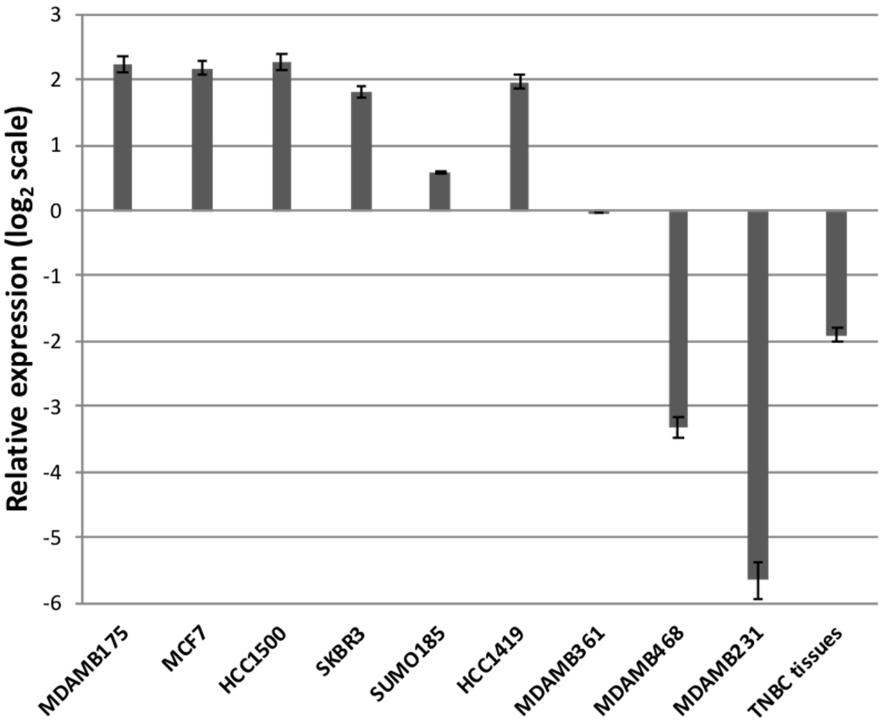

Figure S3. Fold change of expression levels for SELENBP1 mRNA in all breast cancer cell lines compared to MCF-10A cells and in TNBC tissues compared to the normal counteparts, evaluated by $2^{-\Delta\Delta Cq}$ method and reported as $\log_2$ scale. We consider statistically significant the values higher and lower than +1 and −1, respectively.

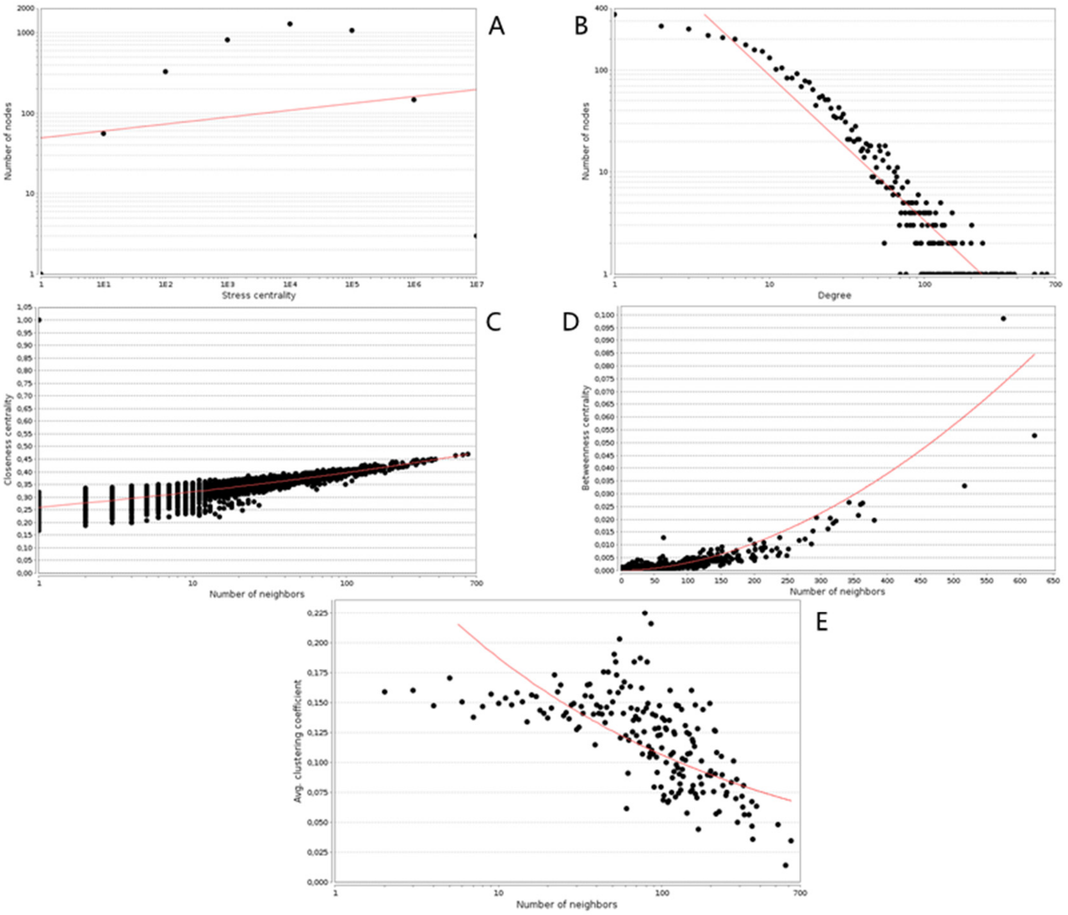

Figure S4. Evaluation of topological properties of TNBC network. (**A**) stress centrality, (**B**) node degree distribution, (**C**) closeness centrality, (**D**) betweenness centrality measure and (**E**) average clustering coefficient.

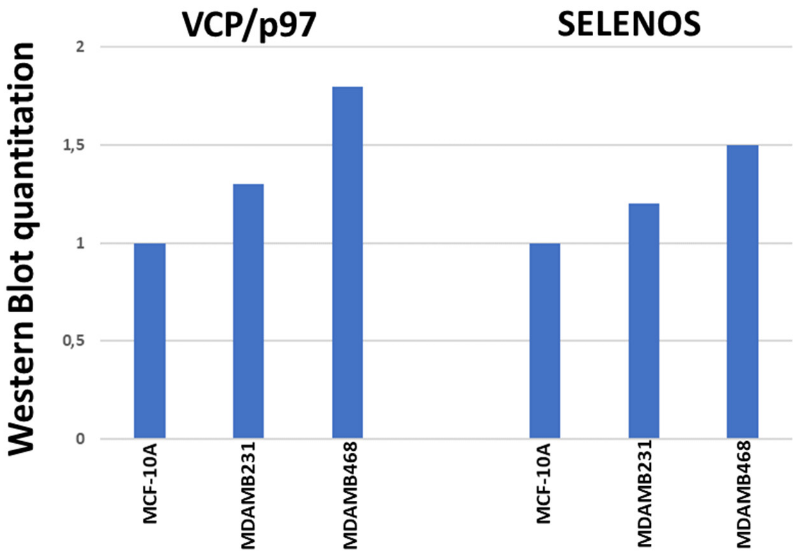

Figure S5. We show as an expression graphthe quantifications performed for VCP/p97 and SELENOS by Western blot plots using IMAGEJ software.

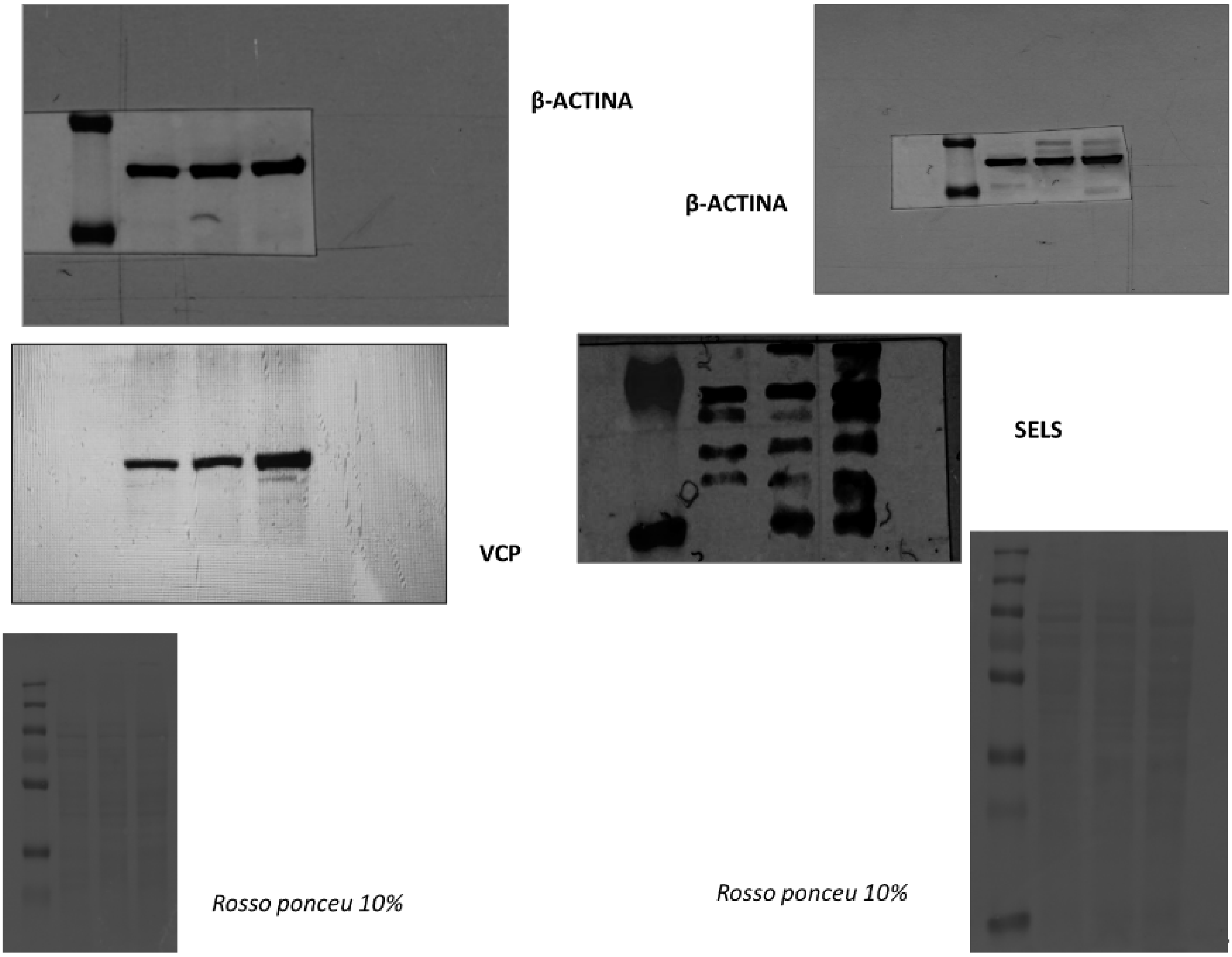

Figure S6. We report the original uncropped Western Blots of Figure 4B.

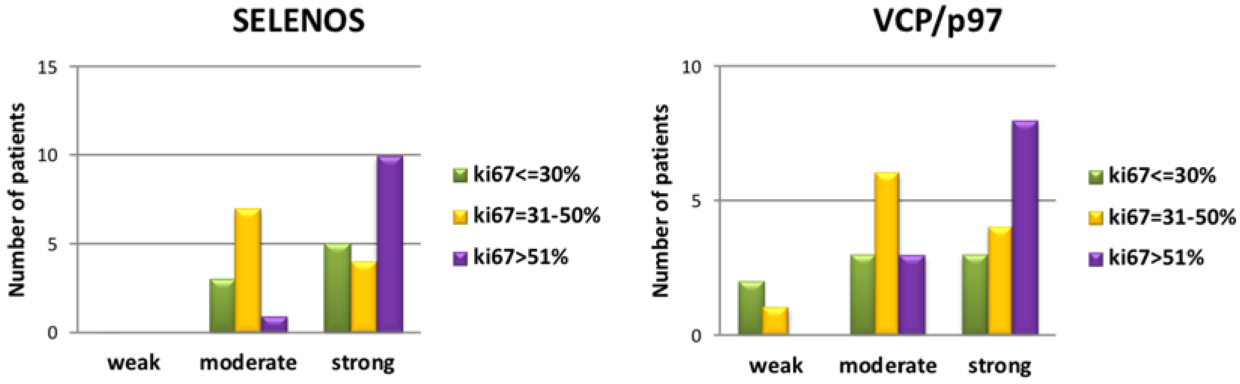

Figure S7. Correlation between SELENOS and VCP/p97 expression and ki67. Number of patients with gradual SELENOS and VCP raise expression (indicated as weak, moderate and strong) are reported respect to different ki67 values.

Table S1. List of primer sequences.

Gene	Melting Temperature (°C)	Sequence 5′→3′
DIO1	59.8	AGCTTACTCTGGCTTTGCCGA (21)
		TATTACCCGTCTTCTCGCCCA (21)
DIO2	59.8	CTTACTCTGGCTTTGCCGAGA (21)
		CAGGATGTTCCGCTTGACTCT (21)
DIO3	59.8	GGTAGTTTCCCCCGCTTGTTT (21)
		TTTAGGTGCTGCTTTGAGGCC (21)
GPX1	59.8	TTATGACCGACCCCAAGCTCA (21)
		ATGTCAATGGTCTGGAAGCGG (21)
GPX2	57.3	GGAGAATGAACCCAAGCGAA (20)
		CAGGTTTGTCACAGCCAGTGAT (22)
GPX3	59.8	TCTCATCCCATGTCCACCATG (21)
		TGCATCCATTTGTGCCAGG (19)
GPX4	59.8	AGAGATCAAAGAGTTCGCCGC (21)
		TCTTCATCCACTTCCACAGCG (21)
GPX6	59.8	CAGAAACCCCACCTCACATGA (21)
		TGCCATGACCTGAATGCACT (20)
MSRB1	59.8	AGCGGCTGTTGCTCCATAACT (21)
		ATTTCAGCATCACCCACCCTC (21)
SELENBP1	61.4	GGGAGGTACATGGTCAGTGG (20)
		GGAAGAGCTGTCCTGTGAGG (20)
SELENOF	59.8	ATCGGAGGCATGCAGAGAGTT (21)
		TCTGCAATCAGGATCCAGCTG (21)
SELENOH	57.9	GGTGGTGGAAGAGTTGAAGAA (21)
		AGGGACACAAAGCTCAGCAT (20)
SELENOI	56.7	AAAGGCCAGGTTCCCAGAA (19)
		CAATCCTGCTGCAGTCCAAGT (21)
SELENOK	57.3	AATCAATCATCTGCGTGGCC (20)
		TGGTCAGCCTTCCACTTCTTG (21)
SELENOM	57.9	TCACGCAGGACATTCCATTCT (21)
		CCTGCACTAGCGCATTGATCT (21)
SELENON	59.8	AGGCAGATGCTCATTGTTCCC (21)
		CCCCAAATCCAGATGCAGACT (21)
SELENOO	59.8	CGGTTGTGTTGCGTGTAGCTT (21)
		TGCACTCGAATGTCGTTCCTC (21)
SELENOP	59.8	TAGGAGCTGATGCTGCCATTG (21)
		ATGTTCTCCTCTGCCCGAAGT (21)
SELENOS	59.8	CAGCTGCTCGACTGAAAATGC (21)
		GCATGCTGTCCCACATTTCAA (21)
SELENOT	57.9	TCAATCCCACACCATCGATCA (21)
		ACAACGAGCCTGCCAAGAAAG (21)
SELENOV	57.9	GTGGATTCGTCATTTCCCATG (21)
		TTTGAGTCTGACTGCCATCCC (21)
SELENOW	59.8	GTTTATTGTGGCGCTTGAGGC (21)
		CCATCACTTCAAAGAACCCGG (21)
SEPHS2	57.3	CGGCTCGCTTTTGTTCTGAA (20)
		TCGCGGCTTGTCAATGATC (19)
TXNRD1	57.9	CACAATTGGAATCCACCCTGT (21)
		GGTTTGCAGTCTTGGCAACA (20)
TXNRD2	57.9	AGGACATTTGCTGGTCGAAGC (21)
		GGAATCCCCTGGAAAAACGTT (21)

TXNRD3	57.9	CCTTTCCCAGTTGCTAGTGC (20)
		GTGCTACACTCTGGGCAACA (20)
VCP	57	GCCTTGAATGAAGTAGGGTAT(21)
		GTTGGGTCTGTTGGTTGC (18)
β-actin	59.8	TCTGGCACCACACCTTCTACAATG (24)
		AGCACAGCCTGGATAGCAACG (21)

Table S2. Functional analysis performed by STRING tool (https://string-db.org/, accessed on 1 May 2020) on five significant selenoproteins (GPX1, GPX4, SELENOS, TXNRD1 and TXNRD3). In the table we report the molecular function, biological Process and Reactome and Kegg Pathways in which these selenoproteins are involved, and the number and the list of involved selenoproteins.

	Number	List of Involved Selenoproteins
**Molecular Function (GO)**		
antioxidant activity	5	GPX1, GPX4, SELENOS, TXNRD1, TXNRD3
glutathione peroxidase activity	2	GPX1, GPX4
oxidoreductase activity	4	GPX1, GPX4, TXNRD1, TXNRD3
thioredoxin-disulfide reductase activity	2	TXNRD1, TXNRD3
protein disulfide oxidoreductase activity	2	TXNRD1, TXNRD3
flavin adenine dinucleotide binding	2	TXNRD1, TXNRD3
electron transfer activity	2	TXNRD1, TXNRD3
**Biological Process (GO)**		
cellular oxidant detoxification	5	GPX1, GPX4, SELENOS, TXNRD1, TXNRD3
respose to oxidative stress	5	GPX1, GPX4, SELENOS, TXNRD1, TXNRD3
cell redox homeostasis	3	SELENOS, TXNRD1, TXNRD3
oxidation-reduction process	4	GPX1, GPX4, TXNRD1, TXNRD3
negative regulation of inflammatory response	2	GPX1, SELENOS
lipoxygenase pathway	2	GPX1, GPX4
response to reactive oxygen species	3	GPX1, TXNRD1, TXNRD3
response to oxygen radical	2	TXNRD1, TXNRD3
cellular response to oxidative stress	3	GPX1, SELENOS, TXNRD1
response to inorganic substance	3	GPX1, TXNRD1, TXNRD3
negative regulation of intrinsic apoptotic signaling pathway	2	GPX1, SELENOS
response to oxygen containing compound	4	GPX1, SELENOS, TXNRD1, TXNRD3
fatty acid derivative metabolic process	2	GPX1, GPX4
electron transport chain	2	TXNRD1, TXNRD3
negative regulation of programmed cell death	3	GPX1, GPX4, SELENOS
multi-organism process	4	GPX1, GPX4, SELENOS, TXNRD3
**KEGG Pathways**		
Glutathione metabolism	2	GPX1, GPX4
**Reactome Pathways**		
Detoxification of reactive oxygen species	2	GPX1, TXNRD1
Synthesis of eicosatetraenoic acid	2	GPX1, GPX4
Metabolism of nucleotides	2	GPX1, TXNRD1
Metabolism of lipids	3	GPX1, GPX4, TXNRD1
**Molecular Function (GO)**		
antioxidant activity	5	GPX1, GPX4, SELENOS, TXNRD1, TXNRD3

Table S3. Detailed analysis of the statistical centrality and topological measures on the networks.

**Statistical Analysis**	
Network centralization	0.144
Network density	0.05
Network heterogeneity	1.729
Characteristic path length	3.166
Clustering coefficient	0.135
Average number of neighbors	21.55
